# Differentiation in poult plumage color of f1 progeny from crosses between white and black indigenous turkeys

**DOI:** 10.1093/tas/txac168

**Published:** 2022-12-23

**Authors:** E O Ewuola, I O Olarinre

**Affiliations:** Animal Physiology and Bioclimatology Unit, Department of Animal Science, Faculty of Agriculture, University of Ibadan, Ibadan, Nigeria; Animal Physiology and Bioclimatology Unit, Department of Animal Science, Faculty of Agriculture, University of Ibadan, Ibadan, Nigeria

**Keywords:** Artificial insemination, poult plumage color, indigenous turkeys

## Abstract

A study assessed the poult plumage color of F1 progeny from artificially inseminated crossings between white and black indigenous turkeys. 72 hens (32 black, 40 white) and 10 toms were used (5 black and 5 white). The turkeys were grouped into four treatments based on the breeding plans: T1 (White toms × White hens), T2 (Black toms × Black hens), T3 (White toms × black hens), and T4 (Black toms × White hens). Semen was harvested from five white toms, pooled, and inseminated into hens in T1 and T3. Semen harvested from five black toms were also pooled and inseminated into hens in T2 and T4. All inseminations were carried out immediately after collection, and each hen received a dose of 0.02 mL. Insemination was done for 2 consecutive days in week 1 and once weekly; eggs were collected and incubated weekly for 12 weeks. Poult plumage colors were monitored and recorded weekly after the first 28 d. Average fertility in each of the treatments 1 (99.63%), 2 (99.81%), 3 (99.84%), and 4 (99.27%) were not significantly (*P* > 0.05) different among the treatments. Hatchability was highest in T2 (72.54%) and least in T1 (57.67%). Percentage white plumage poults in treatments 1, 2, 3, and 4 were 88.73%, 31.61%, 58.15%, and 54.63%, respectively. Percentage black plumage poults in T1, T2, T3, and T4 were 6.97%, 33.04%, 15.26%, and 23.76%, respectively, while 4.30%, 35.35%, 26.57%, and 21.61% were percentage checkered plumage poults in T1, T2, T3, and T4, respectively. There was no significant (*P* > 0.05) difference in the percentage checkered obtained in T2, T3, and T4; percentage of white poult in T3 and T4; percentage of black poult in T3 and T1. The major determinant of poult plumage color concerning quantity was the plumage color of breeder tom semen used for insemination.

## Teaser

No information in the literature provides crossbreeding plans that can be utilized to produce poults of a particular plumage color of choice. This experiment is the first report investigating the poult plumage color of crossbreeding between white and black indigenous turkeys artificially inseminated in Nigeria.

## INTRODUCTION

Domesticated turkeys are usually divided based on plumage color into varieties, six of which are recognized by the America Poultry Association and described in the “standard of perfection.” There is white, which may have flecks or spots of color, and five colored varieties comprising Black, Bronze, Slate, Bourbon Red, and Narragansett. However, the two dominant varieties in Nigeria are white and black plumage. Most indigenous turkeys are bred to have white feathers because their pin feathers are less visible when the carcass is dressed.

Severe feather pecking has been identified as an extremely damaging and prevalent behavior in laying hens. It has acute negative global effects on the egg industry (Hartcher et al., 2016). One of the predisposing factors of feather pecking is plumage color (Bright, 2007; Hartcher et al., 2016).

Similarly, plumage color is second in importance to live weight in affecting consumers’ market preference for chickens in developing countries (Dana et al., 2010). Plumage colors have cultural and religious functions in some African communities (Guèye, 1998; Leulseged, 1998). Certain choices for plumage colors affect the preferences of different geographic markets worldwide (Jiang, 1999; Makarova et al., 2019). Producers, sellers, and intermediary traders of chickens attach high market preference to plumage color and feather distribution (Jiang, 1999). Poult plumage characteristics of crosses between white and black plumage colors have not been adequately predicted, suggesting that qualitative traits with specific characteristics should be carefully identified and considered for the marketability of the indigenous turkeys. Though the bulk of research work on plumage color is on chicken, while a dearth of information still exists on how differences in plumage color influence reproductive performance and poult plumage characteristics in indigenous turkey breeding in Nigeria. It remains unclear whether crosses between white and the black plumage of indigenous turkeys would produce poults of white or black or of other plumage colors. Therefore, this study aimed to assess the poult plumage color of the progenies from the crosses between indigenous turkeys with white and black plumage.

## MATERIALS AND METHODS

All procedures used in this experiment were in line with the guidelines of the University of Ibadan Animal Ethics Committee for research on Poultry in Nigeria. The experiment was carried out at the poultry unit of the Teaching and Research Farm and the Animal Physiology Laboratory, Department of Animal Science, of the University of Ibadan, Nigeria. A total of 82 white and black indigenous turkeys (*Meleagris gallopavo*) aged approximately 8 months old, comprising 10 toms (5-black toms and 5-white toms) and 72 turkey hens (40-white hens and 32-black hens) were sourced locally from a turkey breeder in Ibadan, Oyo State, Nigeria. The birds were differently allotted to treatments based on crosses within and between white and black plumage indigenous turkeys.

The turkeys of two different colors (black feather and white feather) and sexes (Male and Female) were purposively selected and grouped into four mating/crossbreeding plans using 5-white and 5-black indigenous turkey toms and 40-white and 32-black indigenous turkey hens. The four treatment groups were T1 (crosses between 20 white hens and pooled semen from 5 white toms), T2 (crosses between 16 black hens and pooled semen from 5 black toms), T3 (crosses between 20 white hens and pooled semen from 5 black toms), and T4 (crosses between 16 black hens and pooled semen from 5 white toms).

All hens were inseminated weekly according to the method described by Adebisi and Ewuola (2019a). All inseminations were carried out in the evening immediately after semen collection, and each hen received a dose of 0.02 ml undiluted semen as recommended by Adebisi and Ewuola (2019a). The insemination process was done as described by Burrows and Quinn (1937) and modified by Adebisi and Ewuola (2019a). The insemination was done for two successive days only in the first week of the experimental trial and subsequently, once weekly as recommended by Adebisi and Ewuola (2019b) to ensure only introduced sperm cells into the oviduct were stored in it during the period of egg collection. The inseminations were done after 17:00 h in the evening to minimize the presence of an egg in the oviduct.

The day after the second insemination marked the day for the first egg collection. Eggs were collected daily from the second-day post insemination from each treatment group, marked, and stored in egg crates before incubation. Incubation of eggs was done weekly for 10 weeks following hatchery protocols. On day 25 of incubation, egg candling was done, and all candling clears were removed. Eggs with evidence of developing embryo were immediately transferred into the hatcher machine for the egg to hatch to poults on day 28.

All dead embryos were considered fertile. The fertility levels of each treatment flock were calculated as outlined by Sotirov et al. (2002) and recorded in percentage


$$\begin{array}{l} Fertility\;\;\;\left(\%\right)=\frac{number\;\;\;of\;\;\;eggs\;\;\;set}{number\;\;\;of\;\;\;fertile\;\;\;eggs\;}\times 100 \end{array}$$

The hatchability levels per treatment were calculated by Hafez and Hafez (2013) and Wilson et al. (1979) and recorded in percent.


$$\begin{array}{l} Hatchability\;\left(\%\right)=\frac{Number\;\;\;of\;\;\;poults\;\;\;hatched}{Fertile\;\;\;egg\;\;\;at\;\;\;candling}\times 100 \end{array}$$

Poult plumage characteristics were determined by visual appraisal. Poults were classified as white, black, and checkered. The proportion of poults with white, black, or checkered plumage color per treatment was carefully recorded and estimated in percentage.

Data were collected on weekly egg fertility, the hatch of set eggs, and poult plumage classification. The data were subjected to descriptive statistics and analyzed for significance at *P* < 0.05 using the General Linear Model of SAS. The means were separated using Duncan’s multiple range test of the same software.

## RESULTS

The number of settable eggs (%), fertile eggs (%), and poult plumage type (%) are presented in Table 1. Settable eggs in treatments 1, 2, 3, and 4 were 28.60 ± 15.50, 33.10 ± 16.91, 26.90 ± 18.22, and 29.50 ± 18.93, respectively. Fertile eggs in treatments 1, 2, 3, and 4 were 28.40 ± 15.15, 33.00 ± 16.79, 26.80 ± 18.00, and 29.30 ± 18.96, respectively. The number of white poults in treatments 1, 2, 3, and 4 were 14.20 ± 7.54, 7.70 ± 4.37, 9.50 ± 6.49, and 10.90 ± 2.65, respectively Black poults in treatments 1, 2, 3, and 4 were 0.80 ± 1.32,7.50 ± 3.53,3.40 ± 3.24, and 3.90 ± 2.42, respectively. Checkered plumage in treatments 1, 2, 3, and 4 were 0.70 ± 1.25, 9.10 ± 7.69,5.50 ± 5.06, and 4.40 ± 3.17, respectively. The total number of poults in treatments 1, 2, 3, and 4 were 15.70 ± 7.21, 24.30 ± 13.86, 18.40 ± 13.66, and 19.20 ± 13.58, respectively. The average number of settable and fertile eggs was highest and least in treatments 2 and 3, respectively. Treatment 1 had the highest white poults, whereas treatment 2 had the least. There was no significant (*P* > 0.05) difference between treatments 3 and 4 concerning the number of black poults. Treatment 2 had the highest number of black poults compared to other treatments. The number of black poults in treatment 2 was significantly (*P* < 0.05) higher than that of black poults in treatments 1 and 3. Treatment 2 had the highest number of checkered poults. Treatment 1 had the least number of checkered poults. Treatment 2 was significantly (*P* < 0.005) higher than treatment 1 concerning the number of checkered poults. There was no significant difference between the number of checkered poults in treatments 2 and 3 and treatments 1 and 4. The total number of poults was highest and least in treatments 2 and 1, respectively.

**Table 1.** Mean values of numbers of settable eggs (%), fertile eggs (%), white poult (%), black poult (%), checkered (%), and total poult (%) across treatments (Mean ± SD)

Treatments					
Parameters	T1	T2	T3	T4	*P* value
NSE	28.60 ± 15.50	33.10 ± 16.91	26.90 ± 18.22	29.50 ± 18.93	0.88
NFE	28.40 ± 15.15	33.00 ± 16.79	26.80 ± 18.00	29.30 ± 18.96	0.87
White poults	14.20 ± 7.54	7.70 ± 4.37	9.50 ± 6.49	10.90 ± 2.65	0.21
Black poults	0.80 ± 1.32^c^	7.50 ± 3.53^a^	3.40 ± 3.24^b^	3.90 ± 2.42^b^	0.00
Checkered poults	0.70 ± 1.25^c^	9.10 ± 7.69^a^	5.50 ± 5.06^ab^	4.40 ± 3.17b^c^	0.01
Total poults	15.70 ± 7.21	24.30 ± 13.86	18.40 ± 13.66	19.20 ± 13.58	0.48

a, b, and c means along the same column with different superscripts are significantly (*P* < 0.05) different.

SD: standard deviation; T1: white indigenous turkey toms versus white indigenous turkey hens; T2: black indigenous turkey toms versus black indigenous turkey hens; T3: white indigenous turkey toms versus black indigenous turkey hens; T4: black indigenous turkey toms versus white indigenous turkey hens.

Egg fertility (%) and hatchability (%) of crosses between white and black indigenous turkeys are presented in Table 2. Treatment 1 had 99.63 ± 1.17 and 57.67 ± 16.41 as mean values for fertility and hatchability, respectively. Fertility and hatchability in treatments 2, 3, and 4 were 99.81 ± 0.61 and 72.54 ± 13.37; 99.84 ± 0.51 and 67.65 ± 12.52; 99.27 ± 1.53 and 64.82 ± 17.15, respectively. Treatment 3 had the highest fertility compared with other treatments, whereas treatment 4 had the least fertility. Treatment 2 had the highest hatchability percentage, whereas Treatment 1 had the lowest hatchability percentage compared with the other three treatments.

**Table 2.** Fertility and hatchability responses of white and black indigenous turkeys.

Treatment	Fertility (%) (mean ± SD)	*P* value	Hatchability (%) (mean ± SD)	*P* value
T1	99.63 ± 1.17	0.61	57.67 ± 16.41	0.18
T2	99.81 ± 0.61	0.61	72.54 ± 13.37	0.18
T3	99.84 ± 0.51	0.61	67.65 ± 12.52	0.18
T4	99.27 ± 1.53	0.61	64.82 ± 17.15	0.18

SD: standard deviation; T1: white indigenous turkey toms versus white indigenous turkey hens; T2: black indigenous turkey toms versus black indigenous turkey hens; T3: white indigenous turkey toms versus black indigenous turkey hens; T4: black indigenous turkey toms versus white indigenous turkey hens.

The poult plumage color from crosses between white and black indigenous turkeys artificially inseminated is shown in Table 3. The number of white poults in treatment 1 was significantly (*P* < 0.05) higher than the number of white poults in treatment 2. The number of white poults in treatment 1 was also significantly (*P* < 0.05) higher than the number of white poults in treatment 3 and treatment 4. The number of white poults in treatment 3 was significantly (*P* < 0.05) higher than the number of white poults in treatment 2. There was no significant (*P* > 0.05) difference between the number of white poults in treatments 3 and 4. Treatment 1 had the highest white poults, whereas treatment 2 had the least. The number of black poults in treatment 2 was significantly (*P* < 0.05) higher than the number of black poults in treatment 1 as well as treatment 3. The number of black poults in treatment 3 was significantly (*P* < 0.05) higher than the number of black poults in treatment 1. The number of black poults in treatment 2 was significantly (*P* < 0.05) higher than the number of black poults in treatment 4. Treatment 2 had the highest number of black poults, whereas treatment 1 had the least. The number of checkered poults in treatment 2 was significantly (*P* < 0.05) higher than the number of checkered poults in treatment 1. The number of checkered poults in treatment 2 was also significantly (*P* < 0.05) higher than the number of checkered poults in treatment 3 and treatment 4. There was a significant (*P* < 0.05) difference among the number of checkered poults across all treatments. Treatment 2 had the highest number of checkered poults, whereas treatment 1 had the least.

**Table 3.** Poult plumage color variation (Mean ± SD) between white and black turkeys.

Parameters	White poult (%)	Black poult (%)	Checkered poult (%)
Treatments			
T1	88.73 ± 15.63^a^	6.97 ± 11.96^c^	4.30 ± 7.97^c^
T 2	31.61 ± 8.95^c^	33.04 ± 11.33^a^	35.35 ± 13.72^a^
T 3	58.15 ± 23.28^b^	15.26 ± 11.62^bc^	26.57 ± 15.33^ab^
T 4	54.63 ± 9.03^b^	23.76 ± 11.13^ab^	21.61 ± 8.67^b^
*P* value	<0.01	<0.01	<0.01

a, b, and c means along the same column with different superscripts are significantly (*P* < 0.05) different.

SD: standard deviation T1: white indigenous turkey toms versus white indigenous turkey hens; T2: black indigenous turkey toms versus black indigenous turkey hens; T3: white indigenous turkey toms versus black indigenous turkey hens; T4: black indigenous turkey toms versus white indigenous turkey hens.

## DISCUSSION

The lack of significant difference observed between the number of black and checkered poults obtained in crosses between white toms and white hens imply that there could be an equal number of black and checkered poults when white hens are crossed with white toms. In other words, it suggests that the crosses do not favor the poult of black plumage more than the checkered and vice versa. The significant difference observed between the number of white and black plumage poults obtained in crosses between white toms, and white hens indicate that the crosses favor the production of white poults more than the black poults. Crosses between white toms and white hens had the highest number of white poults and the least number of checkered poults, suggesting that the gene responsible for white color was dominant over black color. This result was in consonant with the prediction of plumage color and dominance of white color (Andersson and Purugganan, 2022).

The number of black poults obtained when black hens were crossed with black toms was significantly higher than that of white poults. Interestingly, the number of checkered poults obtained when black hens were crossed with black toms was significantly higher than the black poults and white poults. The number of checkered poults was highest while the number of white poults was the least when black hens were crossed with the black toms, which implies that breeding black turkey toms and black turkey hens would produce black and checkered and white. There was a significant difference between the number of white poults and black poults obtained when black hens were crossed with white toms. Similarly, the significant difference between the number of white poults and checkered poults when black hens were crossed with white toms suggests that breeding white toms and black hens will produce more white and checkered than black poults. There was no significant difference between the number of checkered and white poults obtained when white hens were crossed with black toms. The number of white poults obtained was highest compared to the number of black and checkered poults in crosses between white hens and black toms. The results suggest that breeding turkey toms (white and black) and turkey hens (white and black) of different plumage colors will produce not only white and black poults but also checkered. This finding was in consonant with the inheritance of plumage color in poultry, as reported by some researchers (Bateson et al., 1905; Knox, 1927). They took up the work after the rediscovery of the Mendelian theory of inheritance in 1900. The plumage colors of poults are affected by the plumage color of crosses of parents and other factors such as mutation or because the parents were not completely pure white and black.

## CONCLUSION

Based on the results obtained in this study, more poults with white plumage color were possible if the white or black hen was inseminated with good quality semen from a white tom. Similarly, more checkered poults are possible if black hens were inseminated with good quality semen from black tom. More poult with black plumage will require inseminating the white hens with good quality semen from black toms.

Therefore, a major determinant of poult plumage color was the plumage color of the breeder tom used for insemination.

## Abbreviations

T: Treatment
T1: White Toms versus White Hens
T2: Black Toms versus Black Hens
T3: White Toms versus Black Hens
T4: Black Toms versus White Hens
NSE: number of settable eggs
NFE: number of fertile eggs

## References

[CIT0001] Adebisi, K. A., and E. O. Ewuola. 2019a. Egg fertility and embryo viability of turkey hens inseminated at varied intervals. Livestock Res. Rural Develop. 31.

[CIT0002] Adebisi, K. A., and E. O. Ewuola. 2019b. Fertility response of indigenous turkey hens to semen dosage and oviductal spermatozoa storage/Respuesta Fértil De Pavas Criollas Turcas a Dosis Seminales Y Al Reservorio Oviductal De Espermatozoides. J. Vet. Androl. 4.

[CIT0003] Andersson, L., and M. Purugganan. 2022. Molecular genetic variation of animals and plants under domestication. Proc. Natl. Acad. Sci. U.S.A. 119:e2122150119. doi:10.1073/pnas.2122150119 35858409 PMC9335317

[CIT0004] Bateson, W., E. R. Saunders, and R. C. Punnett. 1905. Experimental studies in the physiology of heredity. In: Reports to the Evolution Committee of the Royal Society. London: The Royal Society of London.

[CIT0005] Bright, A. 2007. Plumage colour and feather pecking in laying hens, a chicken perspective? Br. Poult. Sci. 48:253–263. doi:10.1080/00071660701370483 17578687

[CIT0006] Burrows, W. H., and J. P. Quinn. 1937. The collection of spermatozoa from the domestic fowl and turkey. Poult. Sci. 16:19–24. doi:10.3382/ps.0160019

[CIT0007] Dana, N., T. Dessie, L. H. Van der Waaij, and J. A. van Arendonk. 2010. Morphological features of indigenous chicken populations of Ethiopia. Anim. Genet. Resour. 46:11–23.

[CIT0008] Guèye, E. H. F. 1998. Village egg and fowl meat production in Africa. Worlds Poult. Sci. J. 54:73–86. doi:10.1079/wps19980007

[CIT0009] Hafez, E. S. E., and B. Hafez. 2013. Reproduction in farm animals. Hoboken, NJ: John Wiley & Sons.

[CIT0010] Hartcher, K. M., S. J. Wilkinson, P. H. Hemsworth, and G. M. Cronin. 2016. Severe feather-pecking in non-cage laying hens and some associated and predisposing factors: a review. Worlds Poult. Sci. J. 72:103–114. doi:10.1017/s0043933915002469

[CIT0011] Jiang, X. 1999. Broiler breeding: breeding goals, selection schemes and the usefulness of local breeds for China. Netherlands: Wageningen University and Research.

[CIT0012] Knox, C. W. 1927. The genetics of plumage color in poultry. Ames, IA: Iowa State University.

[CIT0013] Leulseged, Y. 1998. Study on production systems of indigenous and improved poultry in rural areas of North Wollo.

[CIT0014] Makarova, A. V., O. V. Mitrofanova, A. B. Vakhrameev, and N. V. Dementeva. 2019. Molecular-genetic bases of plumage coloring in chicken. Vavilov J. Genet. Breed. 23:343–3–54. doi:10.18699/VJ19.499

[CIT0015] Sotirov, L., S. Dimitrov, and E. Jeliazkov. 2002. Semen lysozyme levels and semen quality in turkeys (*Meleagris gallopavo*) fed with various dietary protein levels. Rev. Med. Vet. 153:815–818.

[CIT0016] Wilson, H. R., N. P. Piesco, E. R. Miller, and W. G. Nesbeth. 1979. Prediction of the fertility potential of broiler breeder males. Worlds Poult. Sci. J. 35:95–118. doi:10.1079/wps19790008

